# Hydrocinnamic Acid and Perillyl Alcohol Potentiate the Action of Antibiotics against *Escherichia coli*

**DOI:** 10.3390/antibiotics12020360

**Published:** 2023-02-09

**Authors:** Mariana Sousa, Ana Cristina Afonso, Lília Soares Teixeira, Anabela Borges, Maria José Saavedra, Lúcia Chaves Simões, Manuel Simões

**Affiliations:** 1 LEPABE—Laboratory for Process Engineering, Environment, Biotechnology and Energy, Faculty of Engineering, Department of Chemical Engineering, University of Porto, 4200-465 Porto, Portugal; 2 ALiCE—Associate Laboratory in Chemical Engineering, Faculty of Engineering, University of Porto, 4200-465 Porto, Portugal; 3 CITAB—Centre for the Research and Technology of Agro-Environmental and Biological Sciences, University of Trás-os-Montes e Alto Douro, 5000-801 Vila Real, Portugal; 4 CEB, LABBELS—Centre of Biological Engineering, Associate Laboratory on Biotechnology and Bioengineering, and Electromechanical Systems, School of Engineering, University of Minho, 4710-057 Braga, Portugal

**Keywords:** antibiotic recalcitrance, plant-based natural product, perillyl alcohol, hydrocinnamic acid, combinatorial therapy, phytochemical-antibiotic interaction

## Abstract

The treatment of bacterial infections has been troubled by the increased resistance to antibiotics, instigating the search for new antimicrobial therapies. Phytochemicals have demonstrated broad-spectrum and effective antibacterial effects as well as antibiotic resistance-modifying activity. In this study, perillyl alcohol and hydrocinnamic acid were characterized for their antimicrobial action against *Escherichia coli*. Furthermore, dual and triple combinations of these molecules with the antibiotics chloramphenicol and amoxicillin were investigated for the first time. Perillyl alcohol had a minimum inhibitory concentration (MIC) of 256 µg/mL and a minimum bactericidal concentration (MBC) of 512 µg/mL. Hydrocinnamic acid had a MIC of 2048 µg/mL and an MBC > 2048 µg/mL. Checkerboard and time-kill assays demonstrated synergism or additive effects for the dual combinations chloramphenicol/perillyl alcohol, chloramphenicol/hydrocinnamic acid, and amoxicillin/hydrocinnamic acid at low concentrations of both molecules. Combenefit analysis showed synergism for various concentrations of amoxicillin with each phytochemical. Combinations of chloramphenicol with perillyl alcohol and hydrocinnamic acid revealed synergism mainly at low concentrations of antibiotics (up to 2 μg/mL of chloramphenicol with perillyl alcohol; 0.5 μg/mL of chloramphenicol with hydrocinnamic acid). The results highlight the potential of combinatorial therapies for microbial growth control, where phytochemicals can play an important role as potentiators or resistance-modifying agents.

## 1. Introduction

Antibiotics are becoming ineffective mainly due to their extensive and inappropriate use, which results in an increased ability of pathogenic microorganisms to survive these antimicrobial agents [1,2,3]. This is attributed to diverse aspects, particularly the excessive and often inappropriate use of antibiotics in human or veterinary medicine that contributes to the spread of antibiotic resistance; the poor hygiene conditions in some parts of the planet; the continuous flow of travelers, the increase in the number of immunosuppressed patients; and the delay in diagnosis of bacterial infections [4,5].

According to the global priority list of pathogens published by the World Health Organization (WHO) [6], certain microorganisms are becoming particularly dangerous for human health. This list divides microorganisms into three priority categories (critical, high, and medium), with *Escherichia coli* being included in the critical priority category [6]. *E. coli* is a human pathogen responsible for healthcare-associated infections, being one of the most widespread species of Gram-negative bacteria [7,8]. In addition, this species is a good example of microorganisms capable of forming biofilms, which generally complicate the treatment of infections [9,10] and have an exacerbating effect on antibiotic resistance [11,12].

The WHO proposes diverse measures to prevent bacterial infections, including searching for new molecules and developing novel antibiotics [6]. Non-nutritive secondary metabolites from plants, known as phytochemicals, have already proved to be efficient in combating antibiotic-resistant strains [13,14]. Such antimicrobial action has been reported for numerous molecules from diverse classes of phytochemicals [14,15]. Another attractive use of active phytochemicals is their combination with antibiotics [16,17]. The use of phytochemicals and plant extracts as resistance-modifying agents (RMAs) is considered an important research topic due to their decreased risk of developing cross-resistance [16,17,18]. The advantages of combining conventional antibiotics with phytochemicals in the treatment of resistant bacteria are mainly because the mechanisms are multi-target and the possibility of interaction between products can modify or inhibit the mechanisms of bacterial resistance [18,19]. In fact, phytochemicals may have to be applied in high concentrations to achieve the intended antimicrobial effect. Therefore, this is the main reason to test combinations between antibiotics and phytochemicals, to develop effective antibacterial therapies without using high concentrations of a certain compound [14,20]. In that sense, RMAs represent a promising strategy for mitigating the spread of bacterial resistance and to contribute to the recycling of old antibiotics, which are generally cheaper and less toxic than some of the recently launched antimicrobials [17,18,19].

This work aimed to study the antimicrobial activity of two phytochemicals, perillyl alcohol and hydrocinnamic acid, as well as to assess their action in the potentiation of chloramphenicol and amoxicillin against *E. coli*. Perillyl alcohol and hydrocinnamic acid are chemically unrelated molecules, mostly studied for their antimicrobial action and remaining to be studied for their combinatorial effects with antibiotics [21,22]. Furthermore, these molecules belong to classes of phytochemicals for which promising antimicrobial activities have already been reported [23,24]. Perillyl alcohol is a monocyclic terpene derived from mevalonate, produced by diverse plants [25]. This molecule has a cyclohexene substituted by a hydroxymethyl group at C1 and a prop-1-en-2-yl group at C4 [26]. Although the exact antimicrobial mechanism of action of perillyl alcohol is still unknown, it is thought to involve cell membrane disruption [21,23]. Hydrocinnamic acid, also known as 3-phenylpropionic acid, belongs to the class of phenylpropanoids and is produced by animals and plants [27]. This phenolic compound has an aromatic ring, a tail with three carbons, and presents a phenyl group at C3 [27,28]. The mechanism of action of hydrocinnamic acid is related to cell membrane damage and inhibition of virulence factors [24,29,30,31]. Regarding commercial antibiotics, chloramphenicol is an amphenicol presenting a broad-spectrum bacteriostatic activity [32]. Chloramphenicol can bind to the bacterial 50S ribosomal subunit, inhibiting protein synthesis [33]. On the other hand, amoxicillin is a β-lactam antibiotic with a bactericidal broad-spectrum effect [34,35]. Briefly, amoxicillin inhibits cross-linkage between the linear peptidoglycan polymer chains that constitute the bacterial cell wall during the exponential growth phase [34,36,37]. These two antibiotics were chosen in order to understand how phytochemicals can potentiate the action of antibiotics belonging to different classes and with distinct mechanisms of action.

## 2. Results and Discussion

### 2.1. Minimum Inhibitory Concentration and Minimum Bactericidal Concentration

The determination of the minimum inhibitory concentration (MIC) and the minimum bactericidal concentration (MBC) is commonly performed to assess the antimicrobial activity of a molecule against planktonic bacteria [38,39,40]. In this study, the lowest MIC was observed for the antibiotics (Table 1), where the MIC for chloramphenicol was 16 µg/mL, a value within the range of these reported for the *E. coli* strains evaluated by Kidsley et al. [41]. The MBC for chloramphenicol was 64 µg/mL, which was in the range of the values reported by Seukep et al. [42] for several *E. coli* strains. In the case of amoxicillin, a MIC of 8 µg/mL for amoxicillin was obtained. Girlich et al. [43] reported a comparable MIC for amoxicillin against the β-lactam-resistant *E. coli* DH10B (encoding the novel β-lactamase TLA-2) [43,44,45]. The MBC for amoxicillin in this study was 8 µg/mL, which is the same value determined by Kalita et al. [46] for amoxicillin against *E. coli* MTCC 40 (whose special features consist of its genetic stock).

Among the phytochemicals, perillyl alcohol had the lowest MIC (256 µg/mL) and MBC (512 µg/mL). MIC and MBC of perillyl alcohol both five times higher were obtained by Silva et al. [47] against *E. coli* ATCC 25922, proposing that the *E. coli* strain used in the present study is more susceptible to perillyl alcohol than the one used by Silva et al. [47]. In addition, phytochemicals can be considered antimicrobial agents if they display a MIC in the range of 100–1000 µg/mL [48,49,50]. Accordingly, perillyl alcohol has potential clinical relevance as an antimicrobial agent. In the case of hydrocinnamic acid, a MIC of 2048 µg/mL and an MBC > 2048 µg/mL were determined. No other studies were found for this exact molecule (3-phenylpropionic acid) with information regarding the MIC value. However, other hydroxycinnamic acids have already been characterized for their antimicrobial activity, with MIC values between 100 µg/mL and 1500 µg/mL against *E. coli* CECT 434 [51]. This suggests that 3-phenylpropionic acid may have weaker antimicrobial activity than other hydrocinnamic acids. Diverse studies propose that many phenolic acids may have strong antibacterial activity [52,53,54,55,56], an effect related to cell membrane damage and inhibition of virulence factors [24,29,30,31].

The values of half maximal effective concentration (EC_50_) [57,58], were also determined using the Combenefit software for chloramphenicol, amoxicillin, perillyl alcohol, and hydrocinnamic acid (Table 2).

### 2.2. Checkerboard Assay

Phytochemicals have been modestly exploited for their ability to increase the susceptibility of pathogens to several drugs and reduce the toxicity of the therapeutic approach through the use of combinations of molecules [17,59,60,61,62]. The checkerboard assay is widely used to evaluate two antimicrobial agents in combination, providing an excellent analysis of bacterial growth inhibition. Furthermore, this method can also be adapted to allow the study of combinations of a greater number of drugs. This is one of the most consolidated methods for assessing combinatorial outcomes [63,64,65,66,67,68]. Here, six dual combinations: chloramphenicol/perillyl alcohol, chloramphenicol/hydrocinnamic acid, amoxicillin/perillyl alcohol, amoxicillin/hydrocinnamic acid, amoxicillin/metronidazole and perillyl alcohol/hydrocinnamic acid; and two triple combinations: chloramphenicol/perillyl alcohol/hydrocinnamic acid and amoxicillin/perillyl alcohol/hydrocinnamic acid, were studied. The assessment of triple combinations of phytochemical/phytochemical/antibiotic using the checkerboard assay was a novelty of this study. Table 3 shows the fractional inhibitory concentration (FIC) index (FICI) and the classification of each combination, considering the interpretation proposed by Stein et al. [69], with some modifications.

Some studies reported the use of plant extracts to potentiate antibiotics against *E. coli*, including chloramphenicol and amoxicillin [70,71,72,73]. Studies combining antibiotics and individual phytochemicals are scarce. Santos et al. [74] provide evidence on how the interactions between amoxicillin with carotenoids and flavonoids (i.e., lycopene, β-carotene, resveratrol, and rutin) can help control infections by *E. coli*, with synergism being reported. The same conclusions were presented by Rao et al. [75] for the combinations of chloramphenicol with the essential oil from *Geophila repens* (L.) I.M. Johnst. Similarly, the present study showed that these combinations may have high potential, as 50% of the dual combinations between antibiotics and phytochemicals resulted in synergism. Moreover, combinations of molecules with similar or the same mechanism of action are usually not particularly effective since they can “muddle” each other even if their combination does not result in antagonism [76]. Here, the combination perillyl alcohol/hydrocinnamic acid seems to be affected by such an event as well as the combination amoxicillin/perillyl alcohol and the triple combinations. In the case of triple combinations, the FICI resulted from the sum of three individual FIC while for dual combinations it only resulted from the sum of two FIC values. Consequently, for the same type and magnitude of interaction between compounds, the FICI for triple combinations is expected to be higher than for dual combinations. In general, the triple combinations were not particularly effective compared to the dual combinations. Regarding the combination amoxicillin/metronidazole, it is known that metronidazole is very often used as an adjuvant of amoxicillin, enhancing its effect against periodontal infections [77,78,79,80]. In this study, the combination amoxicillin/metronidazole was used as a positive control and resulted in a synergistic effect.

**Figure 1:**
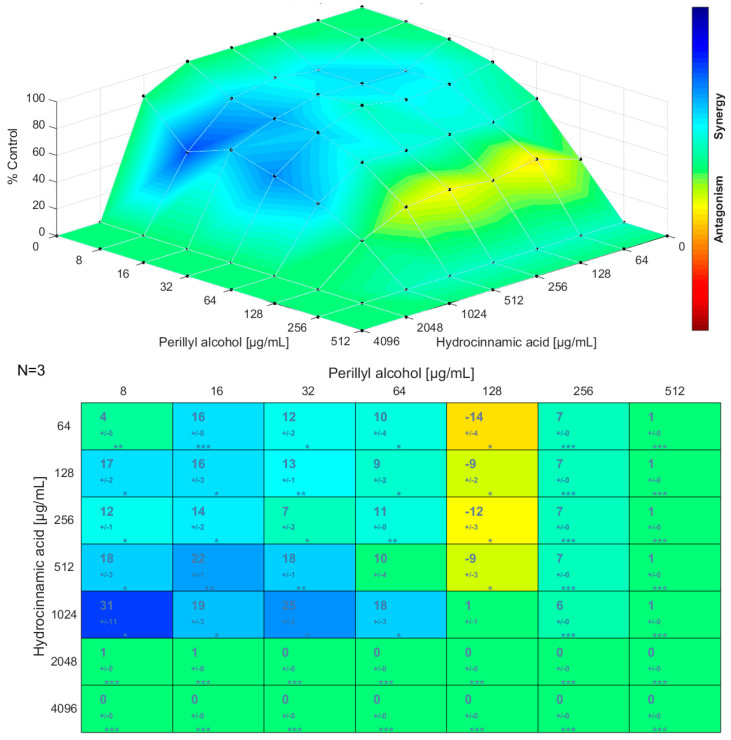
Matrix synergism plot (**top**) and synergism mapped to D-R (**bottom**) for the combination perillyl alcohol/hydrocinnamic acid. The number of stars indicates the level of statistical significance for each score: (*) for a *p*-value less than 0.05, (**) for a *p*-value less than 0.01, and (***) for a *p*-value less than 0.001. The data used are the mean ± standard deviation for three independent experiments.

**Figure 2:**
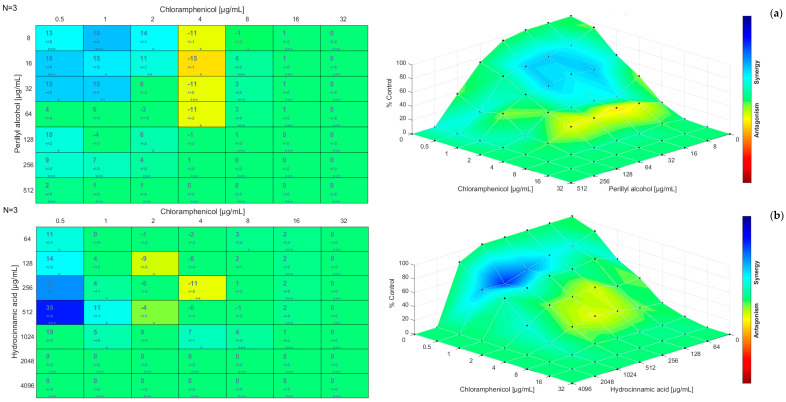
Matrix synergism plot (**left**) and synergism mapped to D-R (**right**) for the combinations: (**a**) chloramphenicol/perillyl alcohol; (**b**) chloramphenicol/hydrocinnamic acid. The number of stars indicates the level of statistical significance for each score: (*) for a *p*-value less than 0.05, (**) for a *p*-value less than 0.01, and (***) for a *p*-value less than 0.001. The data used are the mean ± standard deviation for three independent experiments.

**Figure 3:**
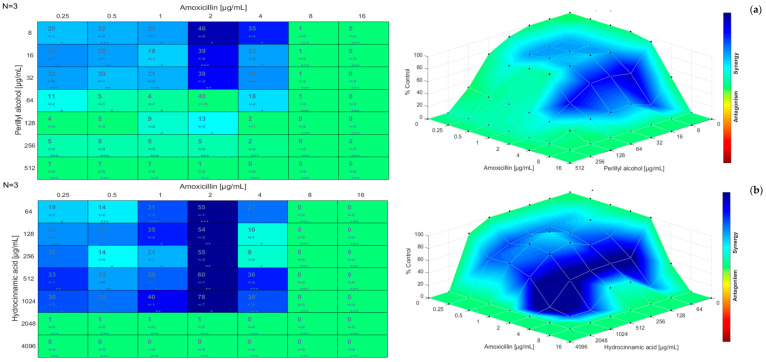

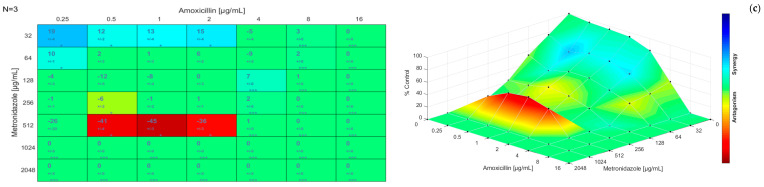
Matrix synergism plot (**left**) and synergism mapped to D-R (**right**) for the combinations: (**a**) amoxicillin/perillyl alcohol; (**b**) amoxicillin/hydrocinnamic acid; (**c**) amoxicillin/metronidazole. The number of stars indicates the level of statistical significance for each score: (*) for a *p*-value less than 0.05, (**) for a *p*-value less than 0.01, and (***) for a *p*-value less than 0.001. The data used are the mean ± standard deviation for three independent experiments.

The results obtained for the checkerboard assay were also analyzed using the Combenefit software. Figure 1, Figure 2 and Figure 3 present the synergism distribution (matrix synergism plot and synergism mapped to D-R, based on the Bliss model) for dual combinations. The single-agent dose response allows to evaluate how the effect of a given molecule varies with the increase of its concentration, until a maximum action is reached [81,82,83]. The matrix synergism plot shows the synergism scores for each combination: stars indicate the level of significance and the number of replicates is reported in the top left corner (in this case, three replicates were performed) [84]. The third-dimension graph (synergism mapped to D-R) shows the percentage of synergism for different concentrations and combinations. In these graphical outputs, blue represents the synergistic combinations, green is used for indifference, and red, orange, and yellow indicate the combinations that resulted in antagonism. Points that are above zero correspond to synergism and points that are below zero correspond to antagonism. Likewise, the points located at the plane or zero synergism percentage correspond to additivity or zero percentage synergism [84,85,86].

Looking at the matrix synergism plot and the synergism mapped to D-R for perillyl alcohol/hydrocinnamic acid (Figure 1), synergism was observed for concentrations of perillyl alcohol between 8 µg/mL and 64 µg/mL and concentrations of hydrocinnamic acid between 64 µg/mL and 1024 µg/mL. For a concentration of perillyl alcohol of 128 µg/mL and concentrations of hydrocinnamic acid between 64 µg/mL and 512 µg/mL, there was a zone of slight antagonism. For a concentration of perillyl alcohol of 256 µg/mL and concentrations of hydrocinnamic acid between 64 µg/mL and 1024 µg/mL, there was a transition zone between synergism and indifference. Indifference occurred for the highest concentrations. The highest synergism score obtained was 31 for 8 µg/mL of perillyl alcohol and 1024 µg/mL of hydrocinnamic acid, and the lowest was −14 for 128 µg/mL of perillyl alcohol and 64 µg/mL of hydrocinnamic acid.

For the combination chloramphenicol/perillyl alcohol (Figure 2a), synergism was verified for lower concentrations of chloramphenicol, up to 2 µg/mL, and for particularly lower concentrations of perillyl alcohol. For a concentration of chloramphenicol of 4 µg/mL and concentrations of perillyl alcohol up to 64 µg/mL, there was a slight antagonism zone. For the higher concentrations, indifference occurred as growth inhibition was observed for these concentrations. The highest synergism score obtained was 19 for 1 µg/mL of chloramphenicol and 8 µg/mL of perillyl alcohol, and the lowest was −15 for 4 µg/mL of chloramphenicol and 16 µg/mL of perillyl alcohol.

When considering the combination chloramphenicol/hydrocinnamic acid (Figure 2b), synergism was verified (although mild for concentrations of chloramphenicol above 0.5 µg/mL) for lower concentrations of chloramphenicol and concentrations of hydrocinnamic acid between 64 µg/mL and 1024 µg/mL. For a concentration of chloramphenicol of 2 µg/mL and 4 µg/mL and concentrations of hydrocinnamic acid between 128 µg/mL and 512 µg/mL, there was a zone of transition between indifference and antagonism. The highest synergism score obtained was 35 for 0.5 µg/mL of chloramphenicol and 512 µg/mL of hydrocinnamic acid and the lowest was −11 for 4 µg/mL of chloramphenicol and 256 µg/mL of hydrocinnamic acid.

Regarding the combination amoxicillin/perillyl alcohol (Figure 3a), a zone of synergism was verified for a concentration of amoxicillin up to 4 µg/mL and a concentration of perillyl alcohol up to 32 µg/mL. For a concentration of amoxicillin of 2 µg/mL and concentrations of perillyl alcohol between 8 µg/mL and 32 µg/mL, a more intense synergism was observed. For concentrations of amoxicillin up to 4 µg/mL and concentrations of perillyl alcohol of 64 µg/mL and 128 µg/mL, there was a zone of transition between synergism and indifference. Indifference was observed for the highest concentrations. The highest synergism score was (46 ± 5) for 2 µg/mL of amoxicillin and 8 µg/mL of perillyl alcohol; the lowest was 0.

In the case of amoxicillin/hydrocinnamic acid (Figure 3b), it was possible to verify a zone of synergism for a concentration of amoxicillin up to 4 µg/mL and a concentration of hydrocinnamic acid up to 1024 µg/mL. It should also be noted that a more intense synergism zone was observed for a concentration of amoxicillin of 2 µg/mL and concentrations of hydrocinnamic acid between 64 µg/mL and 1024 µg/mL. There was indifference for the highest concentrations as growth inhibition was observed. The highest synergism score obtained was 78 for 2 µg/mL of amoxicillin and 1024 µg/mL of hydrocinnamic acid; the lowest was 0.

Finally, for the combination amoxicillin/metronidazole (Figure 3c), a concentration of amoxicillin up to 2 µg/mL and a concentration of metronidazole of 32 µg/mL resulted in a mild synergism zone. There was a strong antagonism zone for concentrations of amoxicillin between 0.5 and 2 µg/mL and for metronidazole at 512 µg/mL. Indifference occurred for the highest concentrations. The highest synergism score obtained was 19 for 0.25 µg/mL of amoxicillin and 32 µg/mL of metronidazole, and the lowest was −45 for 1 µg/mL for amoxicillin and 512 µg/mL for metronidazole.

Overall, after comparing the results obtained for all combinations tested in this study, it was observed that the highest synergism score obtained was 78 for 2 µg/mL of amoxicillin and 1024 µg/mL of hydrocinnamic acid, and the lowest synergism score was −45 for 1 µg/mL of amoxicillin and 512 µg/mL of metronidazole. In addition, it was possible to find a tendency/pattern for the combinations of each antibiotic with perillyl alcohol and hydrocinnamic acid. Not considering the zones of indifference, combinations of amoxicillin with each phytochemical resulted in synergism in a concentration-dependent manner, which was more intense for a concentration of amoxicillin equal to 2 µg/mL. Combinations of chloramphenicol with each phytochemical resulted in synergism mainly at lower concentrations of the antibiotic and a very slight antagonism zone was observed at intermediate or higher concentrations of chloramphenicol. Using Combenefit to evaluate the antimicrobial effect of antibiotic/phytochemical combinations is an innovative approach. In fact, this is a recent tool that allowed us to understand which concentrations of antibiotic and phytochemical in combination can produce higher synergism scores. This software goes beyond the limitation of the checkerboard assay that only allows the calculation of the FICI considering the concentrations for which there is bacterial growth and those for which there is growth inhibition. The distribution of synergism mainly considers what type of interaction occurs until there is growth inhibition and allows determining the best combinations of concentrations [85,86,87,88]. Based on these results, the potential of therapies based on combinations of antibiotics with phytochemicals in the inhibition of *E. coli* growth was clear. This assay has shown that a particular focus should be given to combinations of chloramphenicol/perillyl alcohol, chloramphenicol/hydrocinnamic acid, amoxicillin/hydrocinnamic acid, and amoxicillin/metronidazole while triple combinations were not particularly effective when compared to dual combinations.

### 2.3. Time-Kill Assay

The time-kill assay has been widely used to evaluate the eventual bactericidal effect of single compounds and combinations [89,90,91,92]. This is an excellent test to assess bactericidal effects because it is defined as ≥3 log colony-forming units (CFU) per milliliter decrease between starting and ending points of curves, which is equivalent to 99.9% killing of the initial inoculum [93,94,95]. In this study, a time-kill assay was performed to assess the extent to which each compound and its dual and triple combinations were able to reduce the cellular culturability of *E. coli*.

**Figure 4:**
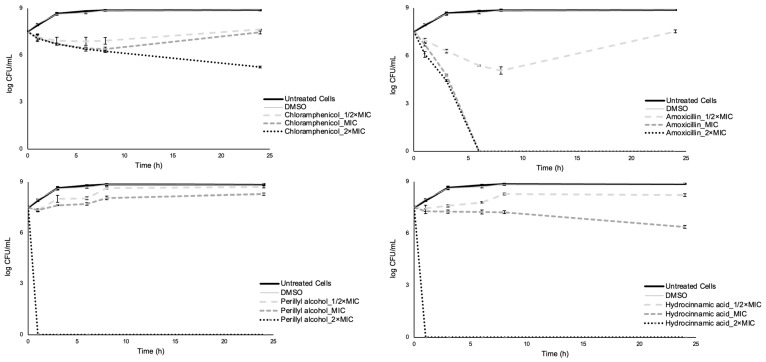
Time-kill curves for individual compounds: chloramphenicol (**top left**), amoxicillin (**top right**), perillyl alchool (**bottom left**), and hydrocinnamic acid (**bottom right**). Two negative controls are presented: *E. coli* and *E. coli* plus the solvent. Values are the mean ± standard deviation for at least two independent experiments.

For single compounds, concentrations equal to 1/2× MIC led to net growth of *E. coli* exposed to both phytochemicals after 24 h, while for the two antibiotics the cell density at 0 h and after 24 h were very similar (*p* > 0.05). For concentrations of MIC, bacterial growth was observed for perillyl alcohol, and a significant reduction of cellular culturability was obtained for amoxicillin (in this case, total reduction) and hydrocinnamic acid. For chloramphenicol, the cell density at 0 h and after 24 h was similar. At 2× MIC concentrations, there was a significant reduction in the bacterial population after 24 h for all the compounds (only not total for chloramphenicol). In that sense, amoxicillin at MIC and 2× MIC and both phytochemicals at 2× MIC exhibited bactericidal effects, causing log CFU/mL reductions higher than 3. Chloramphenicol at all concentrations tested, amoxicillin at MIC, and hydrocinnamic acid at MIC showed a bacteriostatic effect, as the log CFU/mL over time remained relatively stable (<3 log CFU/mL reduction) from the starting value. Finally, perillyl alcohol at 1/2× MIC and MIC and hydrocinnamic acid at 1/2× MIC revealed a modest antimicrobial effect, as the bacteria in the presence of these compounds grew over time to a similar level as the control. These results also reinforce the chloramphenicol status of bacteriostatic antibiotic [96] and amoxicillin as bactericidal [34]. It was also observed that the effect exerted by the phytochemicals was highly dependent on their concentration and the cellular culturability reduction, and the efficacy of the compounds was dose- and time-dependent. In addition, the lowest value of log CFU/mL for chloramphenicol at 1/2× MIC (6.88 after 6 h) and MIC (6.39 after 8 h) and for amoxicillin at 1/2× MIC (5.07 after 8 h) was not obtained after 24 h and the recovery of *E. coli* recovery was evident (Figure 4).

**Figure 5:**
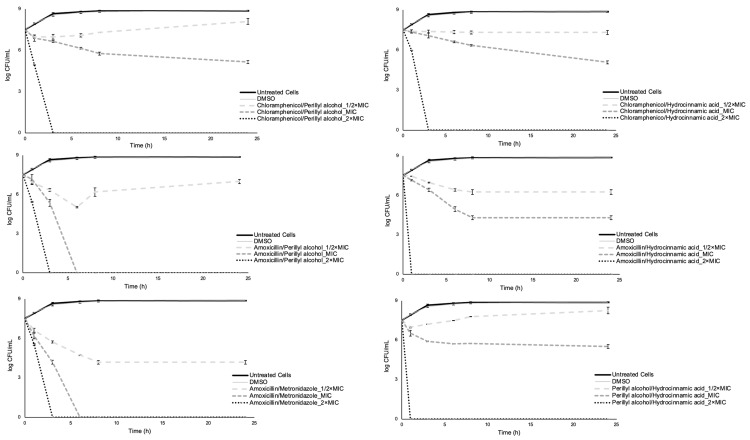
Time-kill curves for dual combinations: chloramphenicol/perillyl alcohol (**top left**), chloramphenicol/hydrocinnamic acid (**top right**), amoxicillin/perillyl alcohol (**middle left**), amoxicillin/hydrocinnamic acid (**middle right**), amoxicillin/metronidazole (**bottom left**), and perillyl alcohol/hydrocinnamic acid (**bottom right**). Two negative controls are presented: *E. coli* and *E. coli* plus the solvent. Values are the mean ± standard deviation for at least two independent experiments.

**Figure 6:**
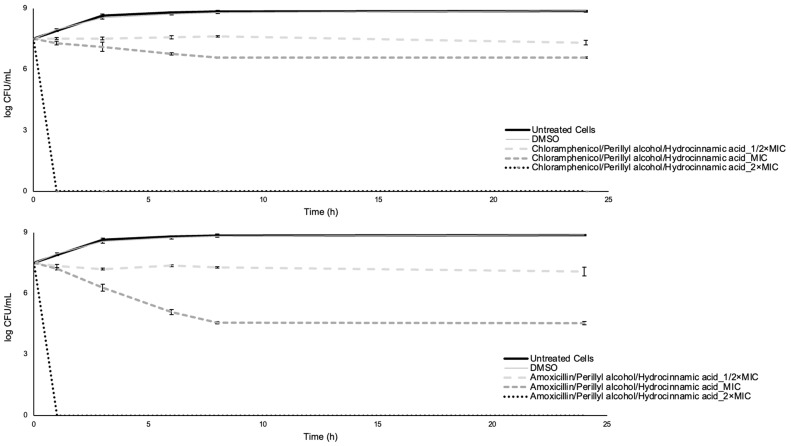
Time-kill curves for triple combinations: chloramphenicol/perillyl alcohol/hydrocinnamic acid (**top**) and amoxicillin/perillyl alcohol/hydrocinnamic acid (**bottom**). Two negative controls are presented: *E. coli* and *E. coli* plus the solvent. Values are the mean ± standard deviation for at least two independent experiments.

Dual and triple combinations were tested for their bactericidal effects. Combinations chloramphenicol/perillyl alcohol at 2× MIC (of each compound), chloramphenicol/hydrocinnamic acid at 2× MIC, amoxicillin/perillyl alcohol at MIC and 2× MIC, amoxicillin/hydrocinnamic acid at MIC and 2× MIC, amoxicillin/metronidazole for all the concentrations, perillyl alcohol/hydrocinnamic acid at 2× MIC and both triple combinations at 2× MIC exhibited bactericidal effect. Combinations chloramphenicol/perillyl alcohol at MIC, chloramphenicol/hydrocinnamic acid at MIC, amoxicillin/perillyl alcohol at 1/2× MIC, amoxicillin/hydrocinnamic acid at 1/2× MIC, perillyl alcohol/hydrocinnamic acid at MIC, and both triple combinations at 1/2× MIC and MIC showed a bacteriostatic effect. Finally, combinations chloramphenicol/perillyl alcohol at 1/2× MIC, chloramphenicol/hydrocinnamic acid at 1/2× MIC, and perillyl alcohol/hydrocinnamic acid at 1/2× MIC revealed little antimicrobial effect. In addition, the lowest value of log CFU/mL for chloramphenicol/perillyl alcohol at 1/2× MIC (6.90 after 1 h), for amoxicillin/perillyl alcohol at 1/2× MIC (5.04 after 6 h), for amoxicillin/metronidazole at 1/2× MIC (4.18 after 8 h), and perillyl alcohol/hydrocinnamic acid at 1/2× MIC (6.98 after 1 h) was not obtained after 24 h, and recovery of cellular culturability was observed (Figure 5 and Figure 6).

Dual and triple combinations were also evaluated through the classification proposed by Zhou et al. [97], with some modifications (Table 4). Considering both scores obtained for combinations with a classification assigned to 1/2× MIC and MIC of each compound, eight combinations resulted in indifference (66.7%), two in additivity (16.7%), and two in synergism (16.7%). Previous studies [98,99] reported synergy by time-kill analysis for combinations of antibiotics and natural compounds (i.e., eugenol, gallic acid, hamamelitannin, epicatechin gallate, epigallocatechin, and epicatechin). In addition, a comparison between the classification attributed to each combination by checkerboard and time-kill assays was possible. For combinations with a classification assigned for 1/2× MIC and MIC of each compound by time-kill, the one for the MIC of each compound was considered for the comparison. Moreover, the percentage of agreement between the checkerboard and time-kill was 87.5%.

Figure S1 (in Supplementary Material) shows the area under the curve (AUC) calculated for each control and treatment, which were used to perform the statistical analysis of time-kill assay results. From the analysis of AUCs (Supplementary Materials Figure S1), it was found that the curve obtained for *E. coli* exposed to 5% (*v/v*) dimethyl sulphoxide (DMSO) was not statistically significantly different from the curve for *E. coli* (*p* > 0.05). Moreover, all treatments were statistically significantly different from controls (*p* < 0.05) except for *E. coli* exposed to perillyl alcohol at 1/2× MIC (*p* > 0.05). The combination amoxicillin/metronidazole was more effective than amoxicillin/perillyl alcohol and amoxicillin/hydrocinnamic acid, at 1/2× MIC and MIC (*p* < 0.05). This assay has shown that a particular focus should be given to combinations of chloramphenicol/perillyl alcohol, chloramphenicol/hydrocinnamic acid, amoxicillin/hydrocinnamic acid, and amoxicillin/metronidazole while triple combinations were not particularly effective when compared to the dual combination.

## 3. Materials and Methods

### 3.1. Bacteria and Culture Conditions

*E. coli* CECT 434 was used in this study. The bacterial strain was stored in cryovials with 30% (*v/v*) glycerol, at −80 °C. In fact, this strain has been previously used in diverse studies to validate the antimicrobial effects of novel molecules [100,101,102,103]. For recovery, bacteria were cultured in Mueller-Hinton Agar (MHA) (Sigma-Aldrich, Merck KGaA, Darmstadt, Germany) plates and allowed to grow for 24 h at 37 °C. The inoculum needed for each assay was prepared by growing *E. coli* overnight in Mueller-Hinton Broth (MHB) (Millipore, Merck KGaA, Darmstadt, Germany), at 37 °C with agitation (150 rpm). The bacteria culture was adjusted to 1.5 × 10^8^ CFU/mL.

### 3.2. Phytochemicals and Antibiotics

Perillyl alcohol (PubChem CID: 369312; Sigma-Aldrich, Merck KGaA, Darmstadt, Germany) and 3-phenylpropionic acid (referred to along this study as hydrocinnamic acid) (PubChem CID: 107; Thermo Fisher Scientific, Acros Organics, Radnor, PA, USA) were tested in this study. Chloramphenicol (PubChem CID: 5959; Sigma-Aldrich, Merck KGaA, Darmstadt, Germany) and amoxicillin (PubChem CID: 33613; Sigma-Aldrich, Merck KGaA, Darmstadt, Germany) were the antibiotics selected. Stock solutions were prepared for all the compounds using DMSO (Avantor, VWR, Radnor, PA, USA). DMSO was used at 5%, and it was verified that this concentration did not affect the growth of *E. coli*. Stock solutions of antibiotics and phytochemicals were prepared and stored at −18 °C.

### 3.3. Minimum Inhibitory Concentration

The MIC of each molecule was determined by the microdilution method according to the guidelines of the Clinical & Laboratory Standards Institute (CLSI) [104], in sterile 96-well flat-bottomed polystyrene tissue culture microtiter plates (Avantor, VWR, Radnor, PA, USA). The phytochemical concentrations tested were between 16 µg/mL and 2048 µg/mL and, in the case of antibiotics were in the range from 0.01 µg/mL to 102.4 µg/mL. Briefly, 10 µL of each dilution was pipetted into the wells of the same column of the microtiter plate. Control wells contained only sterile fresh MHB without bacterial cells, cells with 5% (*v/v*) DMSO (190 μL of cells and 10 μL of DMSO), and only cells (200 μL). Bacteria-free controls constituted by the molecules in sterile fresh MHB were also prepared. A volume of 190 µL of inoculum was added per well. The microtiter plates were then incubated for 24 h at 37 °C and under agitation (150 rpm). The absorbance was measured at 0 h and 24 h using a microtiter plate reader (SPECTROstar^®^ Nano, BMG LABTECH) (at 620 nm). The concentration from which the values at 24 h were equal to or lower than those recorded at 0 h was recorded as the MIC.

### 3.4. Minimum Bactericidal Concentration

MBCs were determined by the drop plate method according to the CLSI guidelines [105]. After the incubation of microtiter plates used for MIC determination, 10 µL were taken from three different wells of each column, gently scraping the bottom of each well with the tip of the micropipette (Eppendorf Research, Merck KGaA, Darmstadt, Germany) to loosen the adhered biofilm, plated in Petri dishes with MHA, and incubated at 37 °C for 24 h. The MBC was then determined visually by observing the lowest concentration from which colonies did not grow.

### 3.5. Checkerboard Assay

The checkerboard microdilution assay was performed according to the CLSI guidelines [104] and Stein et al. [69], with some modifications, using sterile 96-well flat-bottomed polystyrene tissue culture microtiter plates (Avantor, VWR, USA). For dual combinations, concentrations equal to 1/32× MIC, 1/16× MIC, 1/8× MIC, 1/4× MIC, 1/2× MIC, MIC, and 2× MIC of each individual compound, prepared with 5% (*v/v*) DMSO, were combined. One compound was diluted along the *x*-axis, and another was diluted along the *y*-axis. For that, 5 µL of each compound in a certain concentration and 190 µL of inoculum were mixed. The following procedure was performed for triple combinations: two phytochemicals were diluted, one along the *x*-axis and the other along the *y*-axis. Then, one antibiotic was added to the combination of phytochemicals in each well, combining concentrations between 1/4× MIC and 2× MIC of each compound. In this case, 4 µL of each phytochemical, 2 µL of one antibiotic, and 190 µL of inoculum were added to each well. Two controls were performed: 200 µL of sterile fresh MHB and 200 µL of bacterial culture. Microtiter plates were incubated for 24 h, at 37 °C and under agitation (150 rpm). The absorbance of microtiter plates was read at 0 h and 24 h, at 620 nm. Other controls were made in another sterile microtiter plate, consisting of sterile fresh MHB (200 µL), bacterial culture (190 µL) with 5% (*v/v*) DMSO (10 µL), and sterile fresh MHB (195 µL) with each compound (5 µL at 2× MIC). The combination amoxicillin/metronidazole was used as a positive control for a comparison with the results obtained with the other combinations involving amoxicillin since metronidazole is often used as an adjunct to amoxicillin.

### 3.6. Time-Kill Assay

A time-kill assay was performed based on previous studies [97,106,107] with some modifications. Concentrations of 1/2× MIC, MIC, and 2× MIC of each compound were tested, both for assessing the effects of individual molecules and the combinations. The combination amoxicillin/metronidazole was used as a positive control to compare with the results obtained with the other combinations involving amoxicillin since metronidazole is often used as an adjunct to amoxicillin. The individual molecules and combinations being tested were added to bacterial culture in independent sterile microcentrifuge tubes (Avantor, VWR, USA). The total volume was always equal to 1000 µL: 50 µL of a single compound or a combination was added to 950 µL of bacterial culture. Two controls were also evaluated: untreated bacteria and bacterial culture with 5% (*v/v*) DMSO. The tubes were incubated at 37 °C and 150 rpm, for 24 h. Samples were taken after 0, 1, 3, 6, 8, and 24 h of incubation. Then, neutralization was performed by the dilution-neutralization method, using a universal neutralizer: 30 g/L of polysorbate 80 (Avantor, VWR, Radnor, Pennsylvania, USA), 30 g/L of saponin (Avantor, VWR, Radnor, Pennsylvania, USA), 1 g/L of L-histidine (Sigma-Aldrich, Merck KGaA, Germany), 3 g/L of lecithin (Thermo Fisher Scientific, Alfa Aesar, Waltham, Massachusetts, USA), and 5 g/L of sodium thiosulphate (ACP Chemicals Inc., Labkem, Spain) in 0.0025 M phosphate buffer. Then, 100 μL of the sample was added to 800 μL of neutralizer and 100 μL of sterile distilled water. After 10 min at room temperature, ten-fold serial dilutions were performed in sterile NaCl (8.5 g/L) (Avantor, VWR, Radnor, Pennsylvania, USA), from 10^−1^ to 10^−7^. Next, 10 µL were plated on Petri dishes with MHA, following the drop plate method, and were incubated, at 37 °C for 24 h, in the same refrigerated incubator. Finally, CFU was visually counted when the number of colonies ranged from >10 and <100, and expressed as log CFU/mL, according to Equation (1). N is the number of CFU, and SV is the sample volume, 0.01 mL.

(1) $$\mathrm{CFU}/\mathrm{mL}=\frac{N}{\mathrm{SV}\times \mathrm{Dilution}}$$

In this study, a ≥2 log CFU/mL decrease between the combination and its most active constituent was defined as synergism, for 24 h. Likewise, a log CFU/mL decrease between ≥1 and <2 was defined as additivity and a ≥2 log increase was defined as antagonism Indifference was defined as any effect between the limits for additivity and antagonism [97]. Nevertheless, this classification was only applied to treatments for which the CFU counts were above the detection limit, constituting a modification to the classification of Zhou et al. [97]. It was intended not to misinterpret the cases for which the CFU counts were below the detection limit and not to ignore unknown phenomena that may eventually occur in the interaction between compounds.

### 3.7. Classification and Evaluation of Combinations

#### 3.7.1. Interpretation of Checkerboard Assay Results—Fractional Inhibitory Concentration Index

There are four possible results when combining different compounds: Synergism refers to the combination of two or more drugs that results in a greater effect than the sum of the effects for each drug alone [108]. Additivity refers to the combination of two or more drugs that results in an equal effect to the sum of the effects for each drug alone [109]. Indifference refers to the combination of two or more drugs that results in an equal effect on this the most active compound of the combination [110]. Antagonism refers to the combinations of two or more drugs that results in a smaller effect than the sum of the effects for each drug alone [110].

According to Stein et al. [69], FICI = FIC(A) + FIC(B), where FIC(A) is the ratio between the MIC of A in combination and the MIC of A alone (MIC_A_^combination^/MIC_A_^alone^), and FIC(B) is the ratio between the MIC of B in combination and the MIC of B alone (MIC_B_^combination^/MIC_B_^alone^), as presented in Equation (2).

FIC(A) + FIC(B) = (MIC_A_^combination^/MIC_A_^alone^) + (MIC_B_^combination^/MIC_B_^alone^) (2)

For triple combinations, FICI = FIC(A) + FIC(B) + FIC(C), where FIC(A) is the ratio between the MIC of A in combination and the MIC of A alone (MIC_A_^combination^/MIC_A_^alone^), FIC(B) is the ratio between the MIC of B in combination and the MIC of B alone (MIC_B_^combination^/MIC_B_^alone^), and FIC(C) is the ratio of the MIC of C in combination and the MIC of C alone (MIC_C_^combination^/MIC_C_^alone^), according to Equation (3).

FIC(A) + FIC(B) + FIC(C) = (MIC_A_^combination^/MIC_A_^alone^) + (MIC_B_c^ombination^/MIC_B_^alone^) + (MIC_C_^combination^/MIC_C_^alone^) (3)

Applying the classification of Stein et al. [69] with some modifications, FICI ≤ 0.80 corresponds to synergism, 0.80 < FICI ≤ 1.00 indicates additivity, 1.00 < FICI ≤ 4.00 is classified as indifference and FICI > 4.00 is considered antagonism. In this study, 0.8 was considered as the threshold for the synergistic effect between two and three antimicrobial agents based on the classification of Stein et al. [69]; contradicting the limit of FICI < 0.5, commonly used as a limit for synergy between two compounds [111,112,113,114]. Stein et al. [69] performed a three-dimensional synergy analysis (assessing dual and triple combinations by checkerboard), as in this study. Furthermore, the original classification by Stein et al. [69] does not distinguish between indifference and additivity. In the present study, combinations that resulted in a FICI in the range between 0.80 and 1.00 were classified as additive, as a FICI of 1.00 is always equivalent to growth inhibition for the combination of subinhibitory concentrations of all compounds [115].

#### 3.7.2. Interpretation of Checkerboard Assay Results—Fractional Inhibitory Concentration Index

Checkerboard assay results were also analyzed with the Combenefit software version 2.021 (Cancer Research UK Cambridge Institute, Cambridge, UK). Combenefit is open-source software that analyses and classifies combinations based on their effects. The software uses the classic synergism models, namely Loewe, Bliss, and HSA models, to process data [84]. In the present study, the Bliss model was selected to obtain the single agent dose-response and the synergism distribution. The Bliss model is considered appropriate to assess the effect of drugs with independent responses (i.e., when they have distinct modes of action) [116].

### 3.8. Statistical Analysis

Data were analyzed using SPSS version 28.0 (IBM Corp., Armonk, New York, USA). The one-way ANOVA test was followed by Sidak’s multiple comparisons test which is adequate for multiple comparisons. The one-way ANOVA is a parametric test that compares the means of two or more independent groups to determine whether there is statistical evidence that the associated population means are significantly different. It is used when there is one independent variable and one dependent variable. For time-kill curves, AUCs were first calculated, and then the one-way ANOVA test followed by Dunnett’s multiple comparisons test was performed to compare the control with each treatment [117]. The results were presented as mean ± standard deviation. The significance level for the differences was set at *p* < 0.05 and the calculations were based on a confidence level ≥95%. At least two independent experiments, with a minimum of two replicates, were performed for each condition tested.

## 4. Conclusions

With the unprecedented spread of multidrug-resistant (MDR) microorganisms, new, promising molecules and antimicrobial strategies are in great demand. Here, perillyl alcohol and hydrocinnamic acid were studied for their antimicrobial activity against *E. coli*. Interesting MIC values, 256 µg/mL and 2048 µg/mL, were determined for perillyl alcohol and hydrocinnamic acid, respectively. Since one of the main premises of research on antibiotic resistance is to suppress bacterial recalcitrance mechanisms, the use of combined therapies stands out from other options. Therefore, dual and triple combinations between these two phytochemicals and the antibiotics chloramphenicol and amoxicillin were evaluated for the first time. For this, checkerboard and time-kill assays were performed and demonstrated that combinations of chloramphenicol/perillyl alcohol (synergistic by both assays), chloramphenicol/hydrocinnamic acid (additive by both assays), and amoxicillin/hydrocinnamic acid (synergistic by checkerboard and additive by time-kill) were particularly effective. The percentage of agreement between the two assays was 87.5%. In addition, the use of Combenefit was also innovative since it allowed determining the ideal concentrations of each compound in the mixture to produce a synergistic effect. Synergism was found mainly for lower concentrations of antibiotics. The highest synergism score, equal to 78, was obtained for 2 µg/mL of amoxicillin and 1024 µg/mL of hydrocinnamic acid. Moreover, phytochemical concentrations for which synergism was found were also low, allowing researchers to overcome a major problem associated with phytochemical therapies: using high concentrations that can be toxic to human cells. In that sense, it is possible to find new therapies that use low or moderate concentrations of antimicrobial agents without compromising their effectiveness. This study highlighted the effectiveness of possible combination therapy with phytochemicals and antibiotics. Considering the low concentrations for which synergistic effects were observed, the outcomes reported can help recycle antibiotics and mitigate side effects in the human body. The combination of chloramphenicol/perillyl alcohol, being the only interaction classified as synergistic by both checkerboard and time-kill in this study, was particularly promising.

## Figures and Tables

**Table 1:** MICs and MBCs. Values are the mean ± standard deviation for at least two independent experiments.

Compound	MIC (µg/mL)	MBC (µg/mL)
Chloramphenicol	16	64
Amoxicillin	8	8
Perillyl alcohol	256	512
Hydrocinnamic acid	2048	>2048

**Table 2:** EC_50_ for chloramphenicol, amoxicillin, perillyl alcohol, and hydrocinnamic acid.

Compound	EC_50_ (µg/mL)
Chloramphenicol	1.57 ± 0.04
Amoxicillin	3.67 ± 0.28
Perillyl alcohol	96.9 ± 2.1
Hydrocinnamic acid	1208 ± 1

**Table 3:** FICI value and classification of dual and triple combinations. Values are the mean ± standard deviation for at least two independent experiments.

Combination	FICI	Classification
Chloramphenicol/Perillyl alcohol	0.56	Synergism
Chloramphenicol/Hydrocinnamic acid	1.00	Additivity
Amoxicillin/Perillyl alcohol	1.50	Indifference
Amoxicillin/Hydrocinnamic acid	0.75	Synergism
Amoxicillin/Metronidazole	0.63	Synergism
Perillyl alcohol/Hydrocinnamic acid	1.50	Indifference
Chloramphenicol/Perillyl alcohol/Hydrocinnamic acid	1.75	Indifference
Amoxicillin/Perillyl alcohol/Hydrocinnamic acid	1.75	Indifference

**Table 4:** Classification of dual and triple combinations based on time-kill curves. Values are the mean ± standard deviation for at least two independent experiments.

Combination	Δlog CFU/mL	Classification
Chloramphenicol/Perillyl alcohol (1/2× MIC)	0.47	Indifference
Chloramphenicol/Perillyl alcohol (MIC)	−2.29	Synergism
Chloramphenicol/Hydrocinnamic acid (1/2× MIC)	−0.33	Indifference
Chloramphenicol/Hydrocinnamic acid (MIC)	−1.31	Additivity
Amoxicillin/Perillyl alcohol (1/2× MIC)	−0.54	Indifference
Amoxicillin/Hydrocinnamic acid (1/2× MIC)	−1.28	Additivity
Amoxicillin/Metronidazole (1/2× MIC)	−2.67	Synergism
Perillyl alcohol/Hydrocinnamic acid (1/2× MIC)	0.03	Indifference
Perillyl alcohol/Hydrocinnamic acid (MIC)	−0.87	Indifference
Chloramphenicol/Perillyl alcohol/Hydrocinnamic acid (1/2× MIC)	−0.32	Indifference
Chloramphenicol/Perillyl alcohol/Hydrocinnamic acid (MIC)	0.20	Indifference
Amoxicillin/Perillyl alcohol/Hydrocinnamic acid (1/2× MIC)	−0.46	Indifference

## Data Availability

All raw data are available from the corresponding author upon reasonable request.

## Supplementary Materials

The following supporting information can be downloaded at: https://www.mdpi.com/article/10.3390/antibiotics12020360/s1, Figure S1: AUCs values for the time-kill curves.
