# Supplementary material for: Hydrocinnamic Acid and Perillyl Alcohol Potentiate the Action of Antibiotics against Escherichia coli
Source: Antibiotics (Basel). 2023 Feb 9;12(2):360. doi: 10.3390/antibiotics12020360 (PMC9952493; doi:10.3390/antibiotics12020360)
Supplement: Supplementary file 1 [file antibiotics-12-00360-s001.zip › antibiotics-2188501-supplementary.pdf]

Supplementary Materials

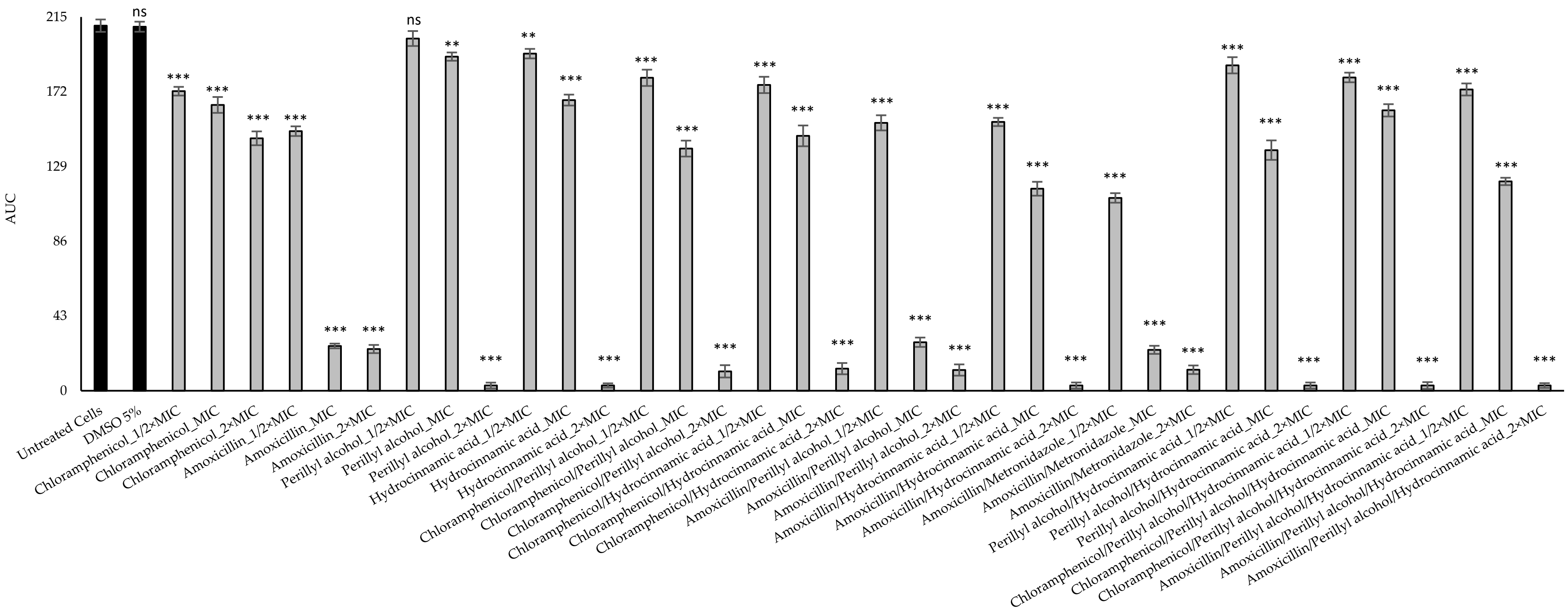

Figure S1. AUCs values for the time-kill curves. The number of stars indicates the level of statistical significance of the difference between untreated cells and *E. coli* exposed to each treatment: (**) for a p-value less than 0.05 and (***) for a p-value less than 0.01. When the statistical difference was not significant, ns was displayed. Values are the mean ± standard deviation for at least two independent experiments.
